# The Role of microRNAs in Pulp Inflammation

**DOI:** 10.3390/cells10082142

**Published:** 2021-08-20

**Authors:** José Luis Muñoz-Carrillo, Silverio Jafet Vázquez-Alcaraz, Jazmín Monserrat Vargas-Barbosa, Luis Guillermo Ramos-Gracia, Israel Alvarez-Barreto, Alejandro Medina-Quiroz, Karla Karina Díaz-Huerta

**Affiliations:** 1 Laboratory of Basic Sciences, School of Odontology, Universidad Cuauhtémoc Aguascalientes, Aguascalientes 20196, Mexico; drajazminvargas@gmail.com (J.M.V.-B.); israel.alvarez.ba@gmail.com (I.A.-B.); alexander.98.m.q@outlook.com (A.M.-Q.); 2 Department of Conservative Dentistry, School of Dentistry, Complutense University, 28040 Madrid, Spain; silveriovazquez.alc@gmail.com; 3 School of Medicine, Universidad Cuauhtémoc Aguascalientes, Jesús María 20908, Mexico; ramos_gracia@hotmail.com; 4 Faculty of Odontology, National Autonomous University of México, México City 04360, Mexico; kardiaz32@hotmail.com

**Keywords:** dental pulp, microRNAs, miR-121, miR-146a, miR-150, miR-152, miR-181, miR-223, miR-30b, miR-Let-7c, pulp inflammation

## Abstract

The dental pulp can be affected by thermal, physical, chemical, and bacterial phenomena that stimulate the inflammatory response. The pulp tissue produces an immunological, cellular, and vascular reaction in an attempt to defend itself and resolve the affected tissue. The expression of different microRNAs during pulp inflammation has been previously documented. MicroRNAs (miRNAs) are endogenous small molecules involved in the transcription of genes that regulate the immune system and the inflammatory response. They are present in cellular and physiological functions, as well as in the pathogenesis of human diseases, becoming potential biomarkers for diagnosis, prognosis, monitoring, and safety. Previous studies have evidenced the different roles played by miRNAs in proinflammatory, anti-inflammatory, and immunological phenomena in the dental pulp, highlighting specific key functions of pulp pathology. This systematized review aims to provide an understanding of the role of the different microRNAs detected in the pulp and their effects on the expression of the different target genes that are involved during pulp inflammation.

## 1. Introduction

The dental pulp is a specialized loose connective tissue, densely innervated and vascularized, enclosed in a rigid environment comprising three mineralized tissues: dentin, enamel, and cementum, which provide it mechanical support and protection against the microbial environment of the oral cavity [1]. The main functions of the pulp are regeneration, repair, as well as defense, through specialized cells of the immune system that help to maintain homeostasis through the formation of dentin, caused by various factors [2], which can induce inflammation of the pulp tissue [3]. Among these factors are microorganisms [4], which can invade the pulp tissue through dental caries, and chemical or mechanical irritation from dental procedures [5]. The pulp inflammatory response is a protective physiological reaction, which aims to eliminate harmful stimuli, with the subsequent initiation of the healing/regeneration process of the pulp tissue [6]. Pulp inflammation is mainly characterized by the dilation of capillaries, the recruitment, and accumulation of inflammatory cells in the pulp tissue [7], which produce and release several inflammatory mediators [8,9], which can influence regeneration and repair of the dentin pulp complex [10].

MicroRNAs (miRNAs) are a class of small endogenous RNA molecules that do not code for proteins, which regulate the transcription of a great diversity of genes through the degradation or repress translation [11]. Currently, the miRBase database lists 38,589 miRNA entries identified in 271 organisms, of which 2812 miRNAs have been identified in humans [12]. MiRNAs play an important role, both in the cellular and physiological functions of all multicellular organisms, as well as in disease [13]. In recent years, several studies have implicated miRNAs in the regulation of the immune system, as well as in inflammatory responses [14], which, together with various pro-inflammatory proteins, regulate their biogenesis, activating rapidly, allowing them to perform different functions in different types of cells, or suppress the expression of different mRNAs, both related to inflammation [15]. Therefore, miRNAs have a fundamental role in the pathogenesis of a wide range of human diseases, such as inflammation, so the alteration in their expression can be a sensitive indicator of these, which could support the diagnosis, prognosis, monitoring, risk, and safety, as possible non-invasive biomarkers [16]. Therefore, the aim of this research is to provide a current review on the understanding of the role of miRNAs as biomarkers and their effects on target genes during pulp inflammation. A specialized systematic search was carried out in databases such as PubMed, through Boolean operators (AND, OR, and NOT), and in ScienceDirect, with the following inclusion terms: dental pulp, pulp inflammation, miRNAs, biogenesis, miRNAs and inflammation, and miRNAs and pulp inflammation.

## 2. Pulp Inflammation

**Figure 1:**
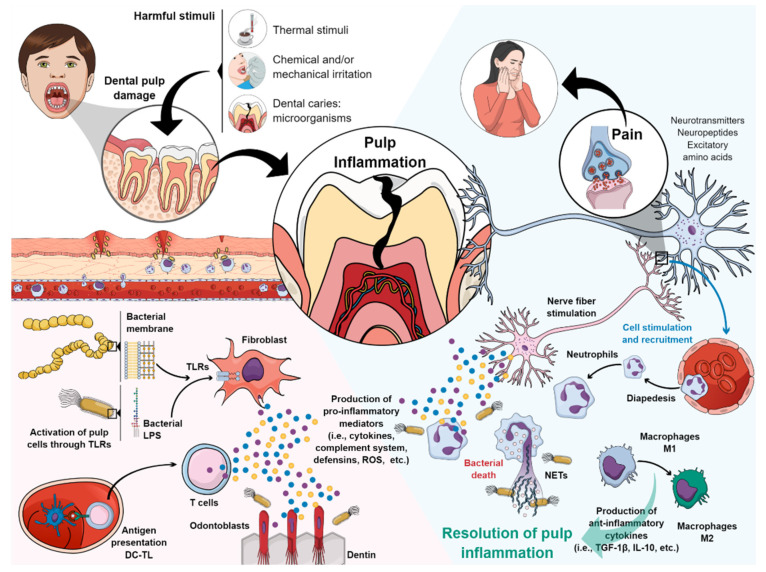
Inflammatory response of the pulp tissue. Explanation in the text. Figure created in MindtheGraph by Muñoz-Carrillo et al., 2021.

Several stimuli, such as thermal, chemical, mechanical, and microbiological, are capable of inducing an inflammatory response in the pulp tissue (Figure 1) [2,3,4,5]. Pulp inflammation initiated, in this case, by microorganisms generates innate mechanisms of immunity that will try to defend the host against invading bacteria [17]. Bacterial components diffuse through the dentin tubules, stimulating odontoblasts and resident cells of the pulp, including fibroblasts, nerve cells, endothelial cells, and stem cells. These cells express many pattern recognition receptors (PRRs), such as Toll-like family receptors (TLRs), which are capable to detect pathogen-associated molecular patterns (PAMPs) such as bacterial lipopolysaccharide (LPS), lipoteichoic acid (LTA), and bacterial DNA [18,19]. Consequently, odontoblasts that have recognized bacterial antigens possess the ability to produce a variety of antibacterial substances to combat invading pathogens [19]. Other cells that recognize antigens, such as pulp fibroblasts, begin local production of complement in the pulp tissue, and the presence of their active molecules (opsonin C3b and CAM) will be involved in the elimination of microbial pathogens [20]. Furthermore, the immature pulp dendritic cells (DC) will mature and influence the process of innate and adaptive immunity, migrating to the lymph nodes, where the antigens will be presented to naive CD4 T cells, which will then differentiate into regulatory CD4 T or effector T helper cells (Th1 or Th2) [7].

A wide range of immune and non-immune cells in the pulp produce and secrete chemokines and cytokines, such as chemokine (C-C Motif) Ligand 2 (CCL2), CXCL1, CXCL2, CXCL8 (IL-8), CXCL10 IL-1α, IL-1β, IL-4, IL-6, IL-10, transforming growth factor (TGF)-1β, and tumor necrosis factor (TNF)-α, which are small glycoproteins (10 to 15 kD) that act as paracrine and autocrine molecular key agents because they regulate the recruitment, extravasation, activation, and differentiation of the immune cells of the pulp tissue [21,22,23]. During these inflammatory events, the nerve fibers of the peripheral nervous system are stimulated, which secrete many molecules, such as classical neurotransmitters (acetylcholine, glutamate, catecholamines), neuropeptides (substance P, calcitonin gene-related peptide, neuropeptide Y, neurokinin A), and excitatory amino acids, which contribute to the induction, sensitization, and maintenance of dental pain [24]. In addition, cytokines, vasoactive molecules, and neuropeptides released locally in the pulp generate signals and gradients that lead populations of immune cells, such as neutrophils, macrophages, and lymphocytes from the bloodstream to the site of inflammation [25].

Extravasated neutrophils in the tissue provide the first line of defense and mediate the destruction of bacteria through a variety of innate immunity mechanisms in terms of their release of antibacterial molecules (ROS and antimicrobial peptides), degrading enzymes of tissue (matrix metalloproteinases) and a bacterial containment and destruction mechanism called extracellular neutrophil traps (NETs) [6,26,27].

When the inflammatory phenomenon persists, the neutrophils recruit monocytes at the site of injury, which differentiate into macrophages; once recruited, and differentiated, the macrophages acquire a pro-inflammatory phenotype, M1 macrophages [28], induced by exposure to pro-inflammatory cytokines, such as IL-1α and IFN-γ [29]. M1 macrophages mediate phagocytosis of bacteria, and cellular debris (efferocytosis) derived from the localized inflammatory process [30]. As pulp inflammation progresses, the synthesis of IL-10 and IL-4 induces polarization of macrophages from an M1 to M2 phenotype [29]. M2 macrophages exert immunomodulatory functions, through the release of anti-inflammatory cytokines, such as TGF-1β and IL-10, which signal tissue remodeling and repair [30,31], leading to the resolution of pulp inflammation. Complement molecules C3a and C5a are also related to tissue-healing/regeneration because they stimulate the proliferation and mobilization of stem cells, fibroblasts, and neural growth [20]. At relatively low levels, cytokines such as TNF-α and TGF-β1, as well as reactive oxygen species (ROS) and bacterial components, can stimulate pulp cell-mediated repair mechanisms. However, when these same molecules are present at higher levels, such as during chronic inflammation, they exert deleterious effects, such as inducing pulp and tissue cell death [32,33].

## 3. Biogenesis of MicroRNAs

Research carried out in the last two decades has revealed different classes of non-coding RNAs (ncRNAs), which are generated from the larger part of the genome that does not encode proteins, but they are capable of regulating gene expression and protein function. The two main classes of ncRNAs are the short ncRNAs (miRNAs) and the long ncRNAs (IncRNAs) [34,35]. MiRNAs are the main class of small noncoding RNAs, which are present in animals, plants, and some viruses [36,37]. MiRNAs have an average length of 18 to 25 nucleotides, which negatively regulate gene expression at the post-transcriptional level, through their binding to the untranslated regions (UTRs) of their target mRNAs, either to suppress the synthesis of proteins or induce mRNA degradation [36,38]. To date, more than 2800 human miRNAs have been reported, of which only a small subset of these are associated with the pathogenesis of a wide variety of diseases, highlighting their critical role in maintaining homeostasis [39].

**Figure 2:**
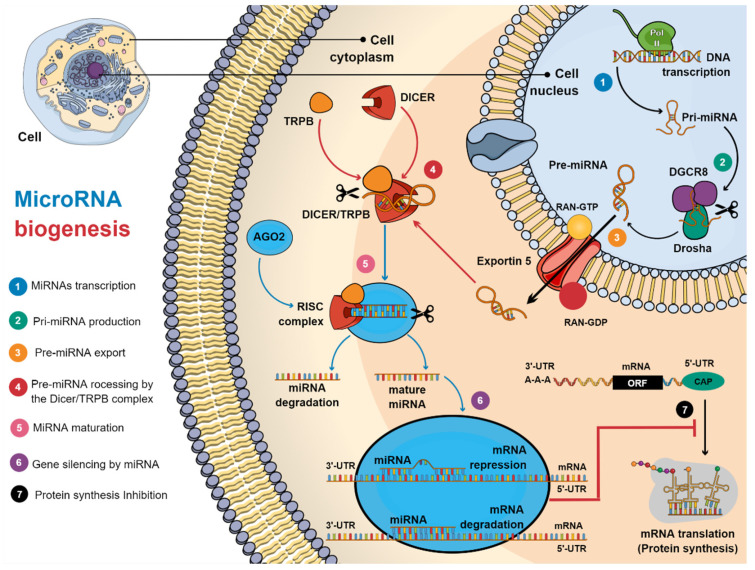
Biogenesis of miRNAs. (1) The transcription of miRNAs by RNA polymerase II (Pol II) produces the primary miRNA (pri-miRNA). (2) The Drosha/DGCR8 complex cuts the pri-miRNA, making the pre-miRNA. (3) Next, exportin-5 promotes nuclear translocation of the pre-miRNA. (4) Once the pre-miRNA has been translocated to the cytoplasm, it is processed by the Dicer/TRPB complex. (5) After cutting, a miRNA:miRNA* duplex is formed, which dissociates, causing the miRNA to mature. (6) Mature miRNA is incorporated into the RISC complex, to mediate gene silencing, either by repression (imperfect matching) or degradation (perfect matching) of target mRNA, inhibiting protein translation (7). Figure created in MindtheGraph by Muñoz-Carrillo et al., 2021.

Currently, two types of miRNAs have been identified, based on their gene processing: (1) intragenic miRNAs, which are processed mainly from introns and, (2) intergenic miRNAs, which are transcribed independently of a host gene, regulated by their promotors [40,41]. Furthermore, miRNAs can be transcribed in clusters, which share similar seed regions [42]. There are two types of miRNA biogenesis, canonical and non-canonical, and the canonical biogenesis pathway is the most predominant, through which miRNAs are processed (Figure 2).

The biosynthesis mechanism of miRNAs is evolutionarily conserved, where miRNAs are transcribed from their genes, and then processed sequentially, involving cleavage mediated by enzymes such as RNA polymerase II (RNA pol II), an RNA-binding protein of the Di George syndrome critical Region 8 (DGCR8), a ribonuclease III enzyme (Drosha), and the Dicer complex [43]. The first incision is made in the cell nucleus from the pri-miRNA, transcribed by RNA pol II, where DGCR8 recognizes the pri-miRNA, to later be incised by the Drosha microprocessor complex [44]. This incision generates a short hairpin of around 60–75 nucleotides [45], called the miRNA precursor (pre-miRNA) [46]. The pre-miRNA is then exported to the cytoplasm via exportin-5 in a Ran-GTP-dependent manner [37]. In the cytoplasm, Dicer, together with TAR RNA binding protein (TRBP), performs the second incision of the pre-miRNA to generate the miRNA duplex [47], which is a double-stranded RNA of approximately 22 nucleotides, that contains a strand of mature miRNA and a strand of passenger miRNA* [48]. The mature miRNA is loaded into argonaut protein (AGO)-2, which promotes the assembly of the protein complex, called RNA-induced silencing complex (RISC), which mediates the recognition of the target mRNA [49]. Mature miRNAs bind to the specific seed region in the 3’-UTR region of the target mRNAs. If base pairing in the seed region is completely complementary to miRNA, the mRNA is targeted for degradation [50]. However, if base pairing is imperfect, it only represses mRNA expression. Both mechanisms result in the inhibition of gene expression at the translational level (protein synthesis) [11].

## 4. Upregulated miRNAs in Pulp Inflammation

In the last decade, a wide variety of studies have been carried out, in which the expression of miRNA in pulp inflammation has been evaluated. Regarding miRNAs that are overexpressed or upregulated in pulp inflammation, it has been reported that they may have different roles during pulp inflammation [51,52] (Table 1).

### 4.1. MiR-21

MiR-21 has an essential role in the resolution of the inflammatory response [102], which acts as a negative regulator of TLR-4 [53], and its overexpression is related to the reduction of IL-6 secretion, the increased production of IL-10 [103] and IL-12 [104], and suppression of the inflammatory phenotype of M1 macrophages [105], implying an anti-inflammatory effect. Nara et al. found that miR-121 was overexpressed in dental pulp cells (hDPC) stimulated with LPS, which was capable to downregulate the production of pro-inflammatory cytokines, such as IL-1α, IL-1β, IL-6, and TNF-α, through repression of TNF receptor-associated factor (TRAF)-6 and programmed cell death (PDCD)-4 mRNAs [54]. Both TRAF6 and PDCD4 are involved in the progression of inflammation, through the activation of the NF-κB pathway [55,56]. Furthermore, in a study by Song et al., it is suggested that overexpression of miR-21 in human dental pulp fibroblasts (HDPF) stimulated with LPS could inhibit the activation of the NF-κB signal pathway [57,105]. These findings indicate the protective role of miR-21 in pulpitis progression.

### 4.2. MiR-146a (miR-146a-5b)

MiR-146a, also called miR-146a-5b, since it is the bioactive mature-5b strand, is a miRNA that is involved in cell proliferation, differentiation, apoptosis, extracellular matrix metabolism, and inflammation [106,107]. MiR-146a targets a variety of molecules, which belong to the NF-κB/pyrin domain-containing protein (NLRP)-3 pathways [108]. MiR-146a overexpression is associated with an anti-inflammatory activity, which is upregulated responding to ligands of TLRs, such as LPS, IL-1β, and TNF-α [58], through the negative regulation of NF-κB [59], targeting its target genes, such as IL-1 receptor-associated kinase (IRAK)-1, TRAF6, and suppressing the mitogen-activated protein kinase (MAPK) pathway [60], in addition to reducing the activity of cyclooxygenase (COX)-2 and 5-lipoxygenase (LO) [61], resulting in a reduction in pro-inflammatory cytokines TNF-α, IL-6, IL-1β, and monocyte chemoattractant protein (MCP)-1 [107,109], preventing cell damage and apoptosis [60]. Regarding miR-146a, its role during pulp inflammation is not yet clear; however, previous studies have shown that miR-146a is upregulated in hDPC stimulated with LPS [110]. A previous study reported that the stimulation of hDPC with LPS induced the overexpression of miR-146a, as well as an increase in the migration of hDPC, through the modulation of the miR-146a-TRAF6/IRAK1 regulatory cascade [62], indicating that miR-146a is essential in enhanced cell proliferation and odontogenic differentiation of hDPC, through modulation of the inflammatory response and regeneration of hDPC [63]. Mo et al. showed that the stimulation of hDPCs with LPS induces miR-146a-5p overexpression, which decreases the protein levels of IRAK1 and TRAF6, in addition to suppressing the expression of IL-6 and IL-8 [64]. These findings suggested that miR-146a has a protective role during pulp inflammation.

### 4.3. MiR-150

Mir-150 has an interesting role in the immune system, since various studies have identified that it has a critical role in the differentiation of B cells [14,15]. During the transition of pro-B to pre-B lymphocytes, miR-150 must normally be downregulated, since its target, the transcription factor c-Myb [65], is necessary for the development of B lymphocytes of the hematopoietic system [66], thus its upregulation inhibits c-Myb, which inhibits the transition of pro-B to pre-B lymphocytes [67]. In addition, a recent study reported that miR-150 is expressed in different tissues and its expression increases in bones, progressively with age, playing an important role in bone homeostasis, in the differentiation and function of osteoblasts, and osteoclasts, since miR-150 serves as a negative regulator of osteoblasts and a positive regulator of osteoclasts, regulating the expression of osteoactivin/glycoprotein nonmetastatic melanoma protein B (Osteoactivin/GPNMB) [68]. It has been reported that miR-150 was significantly upregulated in inflamed human pulps compared to healthy pulps. MiR-150 has as potential target genes interleukin (IL)-6, nuclear factor (κB)-1, Janus kinase (JAK)-2, and interleukin 1 receptor-associated kinase (IRAK)-2 [51]. During pulp inflammation, studies have reported a significant increase of IL-6 [69] at the protein [70,71,72] and mRNA [73,74] level, as well as phosphorylation of IRAK1 [75], expression of NF-κB [76], and activation of the JAK pathway [77]. The production of these pro-inflammatory mediators can be correlated with the degree of inflammation in the pulp tissue, in which miR-150 seems to have an important role as a regulator of inflammatory responses [111], since its overexpression has been shown to have a protective effect on inflammatory responses [112,113].

### 4.4. MiR-223

Studies have shown that miR-223 has an important role in hematopoiesis [78,79], since its overexpression promotes the differentiation of myeloid blast cells [80,81]. Likewise, the overexpression of miR-223 affects cellular differentiation from monocytes to macrophages [82] because miR-223 suppresses TLR-3 [83] and -4 [84] signaling in macrophages and inhibits inhibitory kappa (Iκ)-B kinase (IKK)-α [85] during macrophage differentiation, which are important for the activation and differentiation from monocytes to macrophages [82]. Subsequently, studies showed that miR-223 was expressed in various tissues [114,115], such as bone [116], and that its expression affected cell differentiation, cancer, and inflammation [86]. In this context, miR-223 has an interesting pro-inflammatory role in inflammation, since its overexpression decreases the levels of IKKα and MAP kinase phosphatase (MKP)-5 proteins and induces an increased expression of IL-1β and TNF-α mRNA [87]. During pulp inflammation, the function of miR-223 is still not clear, because, in the study by Huang et al., it was observed that its overexpression in human dental pulp stem cells (DPSCs) significantly increased the levels of dentine sialophosphoprotein (DSPP) and dentine matrix protein (DMP)-1, suggesting that miR-223-3p may be involved in the regulation of odontoblast differentiation during the process of pulpitis repair [88]. However, in the study carried out by Wang et al., it was observed that in the transition from reversible pulpitis to irreversible pulpitis, there was a decrease in the expression of miR-223, playing a role as a negative regulator, involved in the control of the production and secretion of proinflammatory cytokines [89]. These findings show that miR-233 may have a regulatory role depending on the degree of inflammation of the pulp tissue.

### 4.5. MiR-506

Previous studies have reported that miR-506 has an essential function in the regulation of cell differentiation and proliferation [90], in the inhibition of disease development [117], and in cancer progression [118,119,120,121]. Furthermore, studies have reported that miR-506 is associated with the inflammatory response, since its overexpression is capable of activating the immune system [91], favoring the development of various inflammatory diseases [92]. The function of miR-506 in pulp inflammation is not well-known, however, a study carried out by Wang et al. coincides with that previously reported in the literature, since in this study, it was observed that in DPSCs stimulated with LPS, the expression of miR-506 was upregulated, activating the TLR-4 signaling pathway by upregulating sirtuin (SIRT)-1, together with an increase in the expression of pro-inflammatory proteins, such as IL-1β, IL-6, and TNF-α [93]. These findings suggest a pro-inflammatory role for miR-506 [94]; however, more studies are needed to understand the role of miR-506 in the pathogenesis of pulp disease.

### 4.6. MiR-584

Studies have reported that overexpression of miR-584 was capable of suppressing cell proliferation, migration, and invasion, with increased apoptosis in various tumor cells [95,96]. Regarding inflammatory responses, its role is still not well-understood since studies have reported different findings. On the one hand, Zhang et al. found that miR-584 was downregulated in patients with acute respiratory distress syndrome (ARDS), therefore, miR-584 could be involved in the appearance and development of the inflammation of this disease, affecting macrophages and NF-κB [97]. On the other hand, Ouhara et al. reported that *Porphyromonas gingivalis* induced the overexpression of miR-584 in human gingival epithelial cells, favoring the inflammatory response [98]. Regarding the expression of miR-584 during pulp inflammation, it has been reported that it was overexpressed in inflamed human pulps, compared to healthy pulps, having MAPK8 as a potential target gene, which is related to various biological processes, such as apoptosis, cell differentiation, and proliferation, as well as the TLR-4 signaling pathway [51,99]. Furthermore, a recent study reported that miR-584 expression was increased in aged dental pulp tissue compared to young dental pulp tissue [94,100]. These findings show that miR-584 has an important role in pulp tissue homeostasis; however, little information is available, and hence, it is necessary to carry out more studies, which help to better understand the role of miR-584 in pulp inflammation.

### 4.7. MiR-766

Previous studies have reported that miR-766 is an important biomarker in cancer, which contributes to proliferation of cancer cells [122,123,124]. Regarding its role in inflammation, there is only one report to date, which showed that overexpression of miR-766 in human rheumatoid arthritis (RA) fibroblast-like synoviocyte MH7A cells stimulated with TNF-a suppressed the expression of various pro-inflammatory genes, such as IL-1β, IL-6, IL-8, and protein of the matrix metalloproteinase (MMP)-3, thus contributing miR-766, in anti-inflammatory responses, through the indirect inhibition of NF-κB signaling [101]. In pulp inflammation, there is only one study that reports that miR-766 was significantly upregulated in inflamed pulps as compared with normal pulps, whose potential target gene may be heat-shock factor (HSF)-1, which is a heat-shock transcription factor, which is rapidly induced after temperature stress [51,52].

## 5. Downregulated miRNAs in Pulp Inflammation

In the same way as upregulated miRNAs, several studies have also been carried out that have studied the function of miRNAs, when their expression is downregulated during pulp inflammation [51,52] (Table 2).

### 5.1. MiR-Let-7c

MiRNAs belonging to the let-7 family have been shown to play a suppressive role in cancer progression, through direct inhibition of oncogene function [125,126,127]. Furthermore, it has been shown that the overexpression of miR-Let-7c in human dental pulp-derived mesenchymal stem cells (DPMSCs) can inhibit the osteo/odontogenic differentiation of these cells, treated with insulin-like growth factor (IGF)-1 through the JNK/P38/MAPK signaling pathways [128]. On the other hand, studies have shown that miR-Let-7c has an important role in inflammatory responses [99]. Regarding pulp inflammation, a study showed that the expression of miR-Let-7c is altered in inflamed human pulps, whose function is associated with the regulation of the response to mechanical stimuli, through the potential repression of IL-13 [51]. There is evidence that LPS induces downregulation of miR-Let-7c expression in DPSCs, increasing mRNA levels of proinflammatory cytokines such as IL-β and TNF-α [129]. Therefore, it has been shown that the induced overexpression of miR-Let-7c in DPSCs is capable of suppressing the production of pro-inflammatory cytokines, in addition to reducing the infiltration of neutrophils, leading to the restoration of the viability of DPSCs, through inhibition of the activation of the NF-κB signaling pathway, inhibiting phosphorylation of both nuclear factor of kappa light polypeptide gene enhancer in B-cell inhibitor (IκB)-α and inhibitor of nuclear factor kappa-B kinase (IKK)-β, suppressing translocation to the cell nucleus of NF-κB/p65 [130]. Moreover, it has been observed that the overexpression of miR-Let-7c also represses the inflammatory response in DPSCs induced by LPS, through the inhibition of the NF-κB pathway mediated by DMP-1, promoting osteogenic differentiation through the inhibition of high mobility group AT-Hook (HMGA)-2/phosphoinositide-3-kinase (PI3K)/Akt signaling [129]. These findings show that miR-Let-7c may be a therapeutic potential during pulpal inflammation, since the induction of its overexpression leads to an anti-inflammatory effect, through blocking the NF-κB signaling pathway [94].

### 5.2. MiR-125b

Members of the miR-125 family are composed of homologous miRNAs, among which are miR-125a and miR-125b, which are involved in many cellular processes, such as cell differentiation and proliferation, apoptosis, as well as in cancer and other diseases [131]. Normally, miR-125b influences the inflammatory response through the degradation of TNF-α mRNA [132]. However, the expression of miR-125b is downregulated in TLR-4 signaling [15], by the stimulation of LPS in macrophages [133]. On the other hand, it has been reported that miR-125a expression was downregulated during neuroinflammation in mice [134], which was also associated with the development and maintenance of orofacial inflammatory pain [135]. About the role of miR-125a and miR-125b in pulp inflammation, to date, there is no evidence of miR-125b. Regarding miR-125a, there is only one study in which it was observed that the stimulation of DPSCs with TNF-α induces the downregulation of the expression of miR-125b, while the expression of Fyn was upregulated [136]. Fyn is a member of the protein tyrosine kinase *Src* family, and its overexpression is associated with inflammation and odontogenesis [94]. These findings suggest that the induction of miR-125b overexpression in pulpal inflammation can reverse the inflammatory response and improve odontogenic differentiation, through the repression of Fyn [94,136].

### 5.3. MiR-30b

MiR-30b is a member of the miR-30 family, which includes miR-30a, miR-30b, miR-30c, miR-30d, and miR-30e [137]. Previous studies have reported that miR-30b is involved in several processes, such as the development of malignant tumors [138,139,140] and inflammatory responses [141]. In this context, Naqvi et al. reported that miR-30b was found to be downregulated during the differentiation from monocytes to macrophages (Mφ) and from monocytes to dendritic cells (DCs), and that its overexpression was capable to attenuate the phagocytosis by myeloid inflammatory cells, as well as the production of pro-inflammatory cytokines, such as IL-6, IL-12, and TNF-α [142]. Regarding the role of miR-30b in pulp inflammation, there is only one study, which reported that miR-30b was downregulated in pulp tissue, plasma, and saliva, in patients with dental pulpitis, while levels of the IL-6R and IL-6 mRNA remained high, responding to inflammatory processes [143]. These findings show that miR-30b regulates the expression of IL-6R, which can affect the progression of pulpitis through it, so it can be a potential biomarker for the diagnosis of pulpitis.

### 5.4. MiR-152

MiR-152 is one of the members of the miR-148/-152 family, whose physiological functions are related with the control of cell proliferation and differentiation, as well as apoptosis. Besides, a downregulated expression of miR-152 has been frequently detected in many tumors and non-tumor diseases [144,145]. On the other hand, previous studies have reported that miR-152 is a negative regulator of the innate immune response, which affects the antigen presentation capacity of DCs and inhibits the production of pro-inflammatory cytokines, including IL-6, IL-12, IFN-β, and TNF-α [146]. However, its role in inflammation is not yet well-understood, since, on the one hand, studies have reported that in various inflammatory diseases it is downregulated, and that its overexpression is capable of inhibiting cell proliferation, promoting apoptosis [147], protecting against neuroinflammation [148], and reducing the inflammatory process, through the decrease in the production of pro-inflammatory cytokines, such as TNF-α, IL-1β, IL-6, and IL-8 [147]. However, it has recently been observed that overexpression of miR-152 could also aggravate cell apoptosis and the inflammatory response [149,150]. In pulp inflammation, a study showed that miR-152 expression was significantly downregulated in inflamed human pulps, compared to healthy human pulps. This downregulation of miR-152 is associated with the production of IL-6, a potential target gene, which has an important role in pulp inflammation, since it participates in the regulation of the acute inflammatory response and responding to cold, heat, and mechanical stimuli [51,52]. Furthermore, upregulation of miR-152 has also been related to the senescence of DPSCs, through the repression of SIRT7 [151].

### 5.5. MiR-181

The miR-181 family (miR-181a/b/c/d) has previously been shown to inhibit cancer stem cell functions, invasion, and metastasis [152]. Likewise, there is evidence that indicates an essential role of the miR-181 family during inflammation, through the regulation of NF-κB signaling pathways [153,154], since the downregulation of the members of the miR-181 family is associated with the production of pro-inflammatory cytokines, such as TNF-α, IL-6, IL-1β, and IL-8 [155,156], and their overexpression is associated with anti-inflammatory responses [155]. Regarding pulp inflammation, it has been reported that members of the miR-181 family are downregulated in inflamed human pulp [51]. Hence, this family of miRNAs plays an important role during pulp inflammation, since it has been observed that miR-181a regulates the expression of IL-6 [157], miR-181b regulates the expression of CCL8, and it is also associated with the response to thermal and mechanical stimuli [51,158], miR-181c regulates IL-12 expression [159], and miR-181d regulates MMP9 expression [160]. In another study, it was observed that stimulation of human pulp fibroblasts with LPS from *Porphyromonas gingivalis* resulted in a downregulation of miR-181a and an increase in IL-8 expression, identifying IL-8 as another miR-181 target gene [161]. These findings demonstrate that members of the miR-181 family are key in pulp pathology, due to their specific functions.

### 5.6. MiR-204

Previous studies have shown that miR-204 is involved in the development of the retina and eye, as well as in diabetes, many types of cancers, and other disease processes [162]. To date, there is insufficient evidence of the role of miR-204 in inflammation. There is only one study which reported that LPS-induced injury in rat mesangial cells (RMC) induced the overexpression of miR-204, which downregulated the expression of its target gene, IL-6R [163], which shows a possible anti-inflammatory role. Regarding pulp inflammation, likewise, there is only one study in which it was observed that, in children with pulpitis and *Helicobacter pylori* infection in the stomach, the expression of miR-204 was downregulated, with an upregulation of the expression of MMP9, which was identified as a target gene for miR-204 [164]. These findings show that miR-204 can affect inflammatory processes, and that it can be a potential biomarker of pulp pathology and other inflammatory diseases; however, more studies are needed.

### 5.7. MiR-221

Several studies have identified the miR-221 family differentially expressed in different carcinomas, especially breast cancer [165]. Likewise, it has been reported that miR-221 inhibits endothelial cell migration, proliferation, and angiogenesis, by targeting the stem cell factor receptor c-Kit and indirectly regulating the expression of endothelial nitric oxide synthase (eNOS) [166]. Furthermore, miR-221 is considered an antiapoptotic miRNA by targeting the p27 transcript to promote cancer growth and exert antiapoptotic functions [167]. On the other hand, an interesting role of miR-221 has been evidenced in the inflammatory response, since in the inflammation of the colon (experimental colitis), the expression of miR-221 was downregulated [168], while the stimulation of colon epithelial cells with substance P induced the upregulation of miR-221 expression, through the MAPK and NF-κB pathways, which negatively regulated the expression of proinflammatory cytokines [169]. There is not enough evidence regarding the role miR-221 plays in pulpal inflammation, only a previous study reported that its expression was downregulated in hDPC, which would be associated with a variety of biological functions, such as immune response in Toll-like signaling pathways [170]. In addition, miR-221 possibly has an important role in pulp inflammation, through the indirect regulation of eNOS, since various studies have reported that eNOS is expressed in blood vessels [171], and both odontoblasts and endothelial cells of the human pulp [172]. Therefore, its activation by phosphorylation [173] could be associated with the mediation of local vasodilation and cell proliferation in the dental pulp [172].

### 5.8. MiR-410

Aberrant expression of miR-410 has been observed in several types of cancers, suggesting that miR-410 plays an important role in cancer development and progression [174]. Likewise, miR-410 has been shown to mediate inflammatory pathways. Dong et al. reported that miR-410 levels were significantly downregulated in lung tissues with acute lung injury (ALI), where its upregulation markedly suppressed the release of TNF-α, IL- 1β, and IL-6, improving ALI [175]. In another study in mice with osteoarthritis (OA), miR-410 was observed to be markedly downregulated in articular cartilage tissues, as well as in LPS-treated chondrocytes, in OA mice. While the upregulation of miR-410 markedly inhibited the expression of high mobility group box protein (HMGB)-1, a target gene of miR-410, as well as the activity of NF-κB and the production of pro-inflammatory cytokines such as IL-1β, IL-6, and TNF-α [176]. On the other hand, Wang et al. reported that miR-410 expression levels were downregulated in synovial tissues and fibroblast-like synoviocytes (FLSs). Furthermore, it was observed that overexpression of miR-410 significantly reduced the secretion of TNF-α, IL-1β, IL-6, and MMP-9 in human RA fibroblast-like synoviocytes (HFLS-RA), through the suppression of the activation of the NF-κB signaling pathway [177]. Regarding the role of miR-410 in pulp inflammation, there is not much information, however, one study reported that there was a downregulation of miR-410 expression in hDPC stimulated with LPS [178]. These findings show that miR-410 acts as an inflammatory suppressor in the pathogenesis of various inflammatory pathologies, which could be a biomarker in pulpitis; however, more studies are necessary to better understand its role during pulp inflammation.

**Table 2:** Downregulated miRNAs in pulp inflammation.

miRNA	Target Gene	MiRNA Function	Ref
miR-Let-7c	IL-13, IκB-α ^1^, IKK-β^1^, IGF-1R, DMP-1 ^1^, HMGA2/PI3K/Akt ^1^	Osteo/odontogenic differentiation in DPMSCs Response to mechanical stimuli Pro-inflammatory Anti-inflammatory ^1^ Promoting osteogenic differentiation ^1^	[51,94,128,129,130]
miR-25b	TNF-α, Fyn	Development and maintenance of orofacial inflammatory pain	[94,132,135,136]
		Anti-inflammatory and improve odontogenic differentiation	
miR-30b	IL-6R	Attenuate the phagocytosis ^2^ Anti-inflammatory activity ^2^	[142]
miR-152	IL-6, TLR-4, MAPK8, SIRT7	Negative regulator of the innate immune response Protect against neuroinflammation ^3^ Anti-inflammatory ^3^ Response to cold, heat, and mechanical stimuli	[51,52,146,147,148]
miR-181 family	IL-6, IL-2, TGFB1, STAT1 CCL8, MMP9, IL-8	Pro-inflammatory Response to thermal and mechanical stimuli	[51,155,156,157,158,159,160,161]
miR-204	IL-6R, MM9	Anti-inflammatory activity	[163]
miR-221	Stem cell factor receptor c-Kit, p27	Inhibits endothelial cell migration, proliferation, and angiogenesis Antiapoptotic Anti-inflammatory ^4^	[166,167,168,169]
miR-410	HMGB1	Anti-inflammatory ^5^	[176,177]

^1^ MiR-Let-7c overexpression; ^2^ MiR-30b overexpression; ^3^ MiR-125 overexpression; ^4^ MiR-221 overexpression; ^5^ MiR-410 overexpression.

## 6. Clinical Implications and Future Perspectives

Dysfunction of miRNAs has been linked to the development of inflammation. In the case of dental pulp with signs and symptoms indicative of irreversible partial pulpitis due to deep caries, the vital pulp therapy (VPT) using standardized protocols has been suggested to preserve the remaining pulp and reduce the restorative lifecycle of the tooth [179,180]. Modulation of miRNA expression can have beneficial effects on inflammation through the administration of specific miRNA mimics or inhibitors. Thus, modulatory therapies, including modulation of miRNA expression, could have application in clinical VPT protocols. MiRNAs may represent promising therapeutic targets in inflammation. Therefore, the upregulated and downregulated miRNAs in inflamed dental pulp could be of great importance for the therapy of inflammation in the pulp. From a future perspective, it would be interesting to speculate on the possibility that clinical diagnostics could rapidly determine the type and level of inflammation present in the pulp using biomarker pools, such as miRNAs, that can identify and quantify levels of pulp inflammation. However, there are many challenges related to translating this technology into clinical practice, in addition to the cost of developing it and then conducting appropriate clinical trials.

## 7. Conclusions

**Figure 3:**
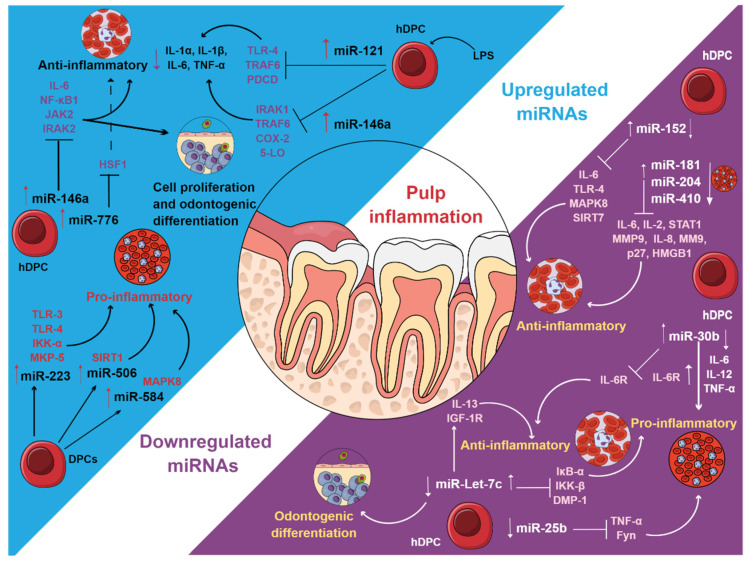
Upregulated and downregulated miRNAs in pulp inflammation. Figure created in MindtheGraph by Muñoz-Carrillo et al., 2021.

The presence and expression of miRNAs can play different roles during pulp inflammation. MiRNAs are overexpressed or downregulated in inflamed pulpal tissues, which are associated with anti-inflammatory and proinflammatory phenomena, cell migration, apoptosis, cell differentiation, and hematopoietic phenomena (Figure 3). MiRNAs may also be negative regulators of the pulp immune response by participating in the regulation of humoral substances, proteolytic substances, and cellular behavior derived from the effect of these miRNAs on several genes during pulp inflammation. A clear understanding of the miRNAs involved in pulp pathology, as well as the target genes and their function, could be useful to be used as possible biomarkers for the diagnosis, prognosis, monitoring, and safety of pulp disease, as well as for future studies of pulp proteomics.

## Figures and Tables

**Table 1:** Upregulated miRNAs in pulp inflammation.

miRNA	Target Gene	MiRNA Function	Ref
miR-21	TLR-4, TRAF6, PDCD	Anti-inflammatory activity Inhibition NF-κB signal pathway	[53,54,55,56,57]
miR-146a	IRAK1, TRAF6, COX-2, 5-LO	Anti-inflammatory activity Suppressing MAPK pathway Cell proliferation and odontogenic differentiation of hDPC	[58,59,60,61,62,63,64]
miR-150	IL-6, NF-κB1, JAK2, IRAK2, c-Myb, Osteoactivin/GPNMB	Differentiation of B cells Differentiation and function of osteoblasts, and osteoclasts Regulator of inflammatory responses	[51,65,66,67,68,69,70,71,72,73,74,75,76,77]
miR-223	TLR-3, TLR-4, IKK-α, MKP-5	Granulopoiesis and monocyte activation Cell differentiation Pro-inflammatory Pulpitis repair	[78,79,80,81,82,83,84,85,86,87,88,89]
miR-506	SIRT1	Cell differentiation and proliferation Activating the immune system Pro-inflammatory	[90,91,92,93,94]
miR-584	MAPK8	Suppress cell proliferation, migration, and invasion Pulp tissue homeostasis Favoring the inflammatory response	[51,95,96,97,98,99,100]
miR-766	HSF1	Inflammatory gene suppressor Temperature stress response	[51,52,101]

## Data Availability

Not applicable.
